# The Honors of War

**Published:** 1851-11

**Authors:** 


THE HONORS OF WAR.

South Carolina has recently done herself the honor of rewarding the medical services of one of her medical men, and it is so rarely the case that politicians pay any attention to the acts of medical servants of the public that we take peculiar pleasure in recording the noble conduct of South Carolina, conduct that confers more honor on her than on the recipient of her testimonial to merit.

Dr. C. J. Clark was physician to the South Carolina regiment in the Mexican war, and displayed the virtues of his profession in such an eminent degree, that South Carolina has presented him with "a gold medal, weighing two and half ounces, upon one side of which is a representation of the landing at Vera Cruz; and on the other, the South Carolina 'coat of arms.'" South Carolina is the only State in the

Union we believe, that has thought it worth while to remember that there were medical men engaged in the service of the war. The medical men of Kentucky rushed zealously to the fields of strife for the purpose of ameliorating the horrors of war by their professional skill and knowledge, but their names have never been mentioned by the State authorities, and no species of honor has been awarded to them.

It seems that our confrére of the New Orleans Medical and Surgical Journal rendered the government some service, professionally, by three months' labor on the Rio Bravo. He was noticed, not by Louisiana, but by the General Government, and Dr. Hester expresses the hope that Dr. Clark will not feel envious of the honors conferred on his war-services. Dr. Hester says: "In turning over to the Quartermaster at this place the medicine and hospital stores in our charge, one or two silver-washed catheters were missing; these were promptly reported to head quarters, and in due time a bill of $40 was returned to us, claiming indemnity for that which was loaned and used up in the service of the country. From that day to this our thirst for military glory has been on the wane, and we wish henceforward to be regarded as an uncompromising advocate for the 'Peace Congress.'" B.

